# Mind as a Behavioral Inhibition Network

**DOI:** 10.3389/fpsyg.2020.00832

**Published:** 2020-05-05

**Authors:** Toru Moriyama, Kohei Sonoda, Hanna Saito, Masao Migita

**Affiliations:** ^1^Faculty of Textile Science, Shinshu University, Ueda, Japan; ^2^Research Organization of Science and Technology, Ritsumeikan University, Kusatsu, Japan; ^3^Graduate School of Interdisciplinary Information Studies, The University of Tokyo, Tokyo, Japan; ^4^Faculty of Education, Shiga University, Otsu, Japan

**Keywords:** behavioral generation module, behavioral inhibition network, emergent behavior, mind, unpredictability

## Abstract

This study aimed to propose to add a new perspective on what may create the impression of “mind” in other beings. The conventional is perspective is that when we observe mental activities in animals, this creates in us the impression that they have a mind. On the other hand, the authors’ proposal is that when we observe unpredictable activities in living beings, this creates in us the impression of mind. This “unpredictability” is a characteristic product of all living things and is not limited to animals. In response to this additional perspective of mind, we assumed that the following questions would arise, “Is mind as the source of unpredictability an imaginary thing? Does it really exist?” To answer this question, a conceptual model of mind was proposed, and its validity was investigated by introducing studies on the relationship between animals’ unpredictability and emergent behavior. In section “Animal Mind as a Behavioral Inhibition Network,” we examined the question from the perspectives of comparative psychology, ethology, and neurophysiology. As a result, we obtained the hypothesis that every animal can have a “behavioral inhibition network” and that this corresponds with the source of unpredictability. The function of the behavioral inhibition network is to create “unpredictable behavior.” It makes an observer facing the animal feel unpredictability of the animal. However, unpredictable behavior may arise from exogenous factors such as congenital malfunction in the mechanism to generate an innately acquired behavior, as well as environmental disturbances. Therefore, in the section “Innate and Emergent Behavior of Animals,” we introduce studies where unpredictable behavior seems to occur endogenously. In these studies, various animal species were examined in unexperienced problem-solving tasks that could not be solved by innately acquired behaviors. As a result, each animal solved the problem by generating unpredictable behaviors with high frequency. Such biologically significant unpredictable behaviors are referred to as “emergent behaviors.” In the section “Discussion,” we investigate whether the behavioral inhibition network matches the mind that one experiences in their daily life. Finally, toward a science of universal mind, we introduce experimental results suggesting the possibility that plants and materials such as stones have a similar structure to a behavioral inhibition network.

## Introduction

What is the mind? It may be one of the most difficult questions ever encountered. Cambridge dictionary (2019) explains the meaning of mind as, “the part of a person that makes it possible for him or her to think, feel emotions, and understand things.” Additionally, one of the most popular Japanese dictionaries, Kojien (2018, p. 1043), poses that, “mind is the source of human mental activity or the activity itself; the cosmos of knowledge, emotion, and will (translated by authors).” By referring to these Western and Eastern definitions, the authors offer a dictionary meaning of mind as “a part of a person that makes mental activity.”

Once one defines the dictionary meaning of mind, the following question may arise, “Do animals have mind?” The answer is tentatively “yes,” as there are a lot of experimental results that have illustrated the mental activities of animals, such as thinking, feeling emotions, and understanding things. For example, a famous parrot named Alex could talk with humans (Pepperberg, 2002); New Caledonian crows can use tools (Hunt, 1996); zebra fishes feel fear (Yoshida and Hirano, 2009); octopuses have the capacity for observational learning (Fiorito and Scotto, 1992); and a single cell organism, slime mold, can solve a maze (Nakagaki et al., 2000).

These examples suggest that all animal species have mind, as mental activity, in varying degrees. This idea is the same as “mental continuity,” which is the basic premise in comparative psychology (Papini, 2002). The concept of mental continuity was proposed originally by Charles Darwin (Darwin, 1871). In his book, *Descent of Man*, he described that, “Nevertheless the difference in mind between man and the higher animals, great as it is, certainly is one of degree and not of kind. We have seen that the senses and intuitions, the various emotions and faculties such as love, memory, attention, curiosity, imitation, and reason, etc. of which man boasts, may be found in an incipient, or sometimes even in a well-developed condition in the lower animals” (p. 101).

In this way, although comparative psychology and related scientific fields have contributed to the growing belief that allows for the existence of mind for all animal species in terms of mental continuity, and further clarified the difference in mind between human and animal, they have not approached the “essential” meaning of mind that we usually feel in our daily life when we contact animals. What is, then, the essential meaning of mind? The authors propose that it is “the source of unpredictability.”

When we meet animals, including humans, face to face, we usually feel a little anxious because their behavior is always unpredictable. For example, Hinde (1982) reported that small male birds, chaffinches, show short-term fluctuations in their reproductive behavior. He described in his book that, “For a while they will sing from conspicuous song posts and attack any other male who encroached on their territories, a few minutes later they may feed alongside other birds in a flock. Such short-term changes in behavior imply short-term changes in internal state-changes that are unlikely to be due to short-term changes in hormone concentration. They may be described as changes in “mood” (p. 27–28).

In the case of slime mold solving a maze, when the amount of the organism or the atmospheric humidity is very high, the plasmodial tube may extend over the wall of the maze and the extra connections across the wall occasionally lead to a path shorter than the normal shortest route (Nakagaki et al., 2001). These examples that illustrate unpredictability of animal behavior allows one to feel the “unpredictability” of animals and further allows for the existence of “mind” inside them.

In the section “Animal Mind as a Behavioral Inhibition Network” of this paper, the authors propose a conceptual model of mind as “behavioral inhibition network” as the scientific approach to mind that one feels in our daily life. Next, we explain the important function of a behavioral inhibition network, “generation of unpredictable behavior,” which makes us feel unpredictability in animals.

In the section “Innate and Emergent Behavior of Animals,” to demonstrate the significant function of a behavioral inhibition network for animals’ survival, the authors report on some experimental results that illustrate how the capacity to generate unpredictable behavior helps animals create “emergent behavior” to survive in unexperienced situations.

In the section “Discussion,” the authors investigate whether behavioral inhibition network matches the mind that one feels in their daily life. Finally, toward a science of universal mind, the authors introduce experimental results suggesting that plants and materials such as stones have a similar structure to a behavioral inhibition network.

## Animal Mind as a Behavioral Inhibition Network

### The Behavioral Generation Module (BGM) and Its Network

In most invertebrates and vertebrates, the nervous system plays a dominant role in the generation of behaviors. In terms of function, it consists of three systems: a sensory system that accepts and processes sensory stimuli from inside and outside the body; a motor system that gives instructions in appropriate time series to the muscles; and an integrating system that connects the sensory and the motor systems, integrates and interprets the input information, and decides what kind of reaction to do (Delcomyn, 1998). One of the main functions of the integrating system is the motivation that processes intrinsic drives (e.g., hunger and sexual maturation) and leads animals to express appropriate innate behaviors (e.g., feeding and mating). The nervous system is also responsible for more simple reactions (e.g., reflex) and more complex behavior (e.g., learning).

On the one hand, a complex nervous system such as the brain seems to be necessary to generate well-controlled behaviors; on the other hand, animals with a simple nervous system also show the same kind of behaviors. For example, starfish demonstrates highly organized behaviors such as body righting (Reese, 1996) and obstacle avoidance (Migita et al., 2005). Additionally, protozoan ciliate, which does not have a nervous system, controls swimming and learns shape and size of an arena (Kunita et al., 2016). These reports suggest that animals, with or without a nervous system, have mechanisms to generate well-controlled behaviors that consists of the three systems, sensory, motor, and integrating.

**FIGURE 1:**
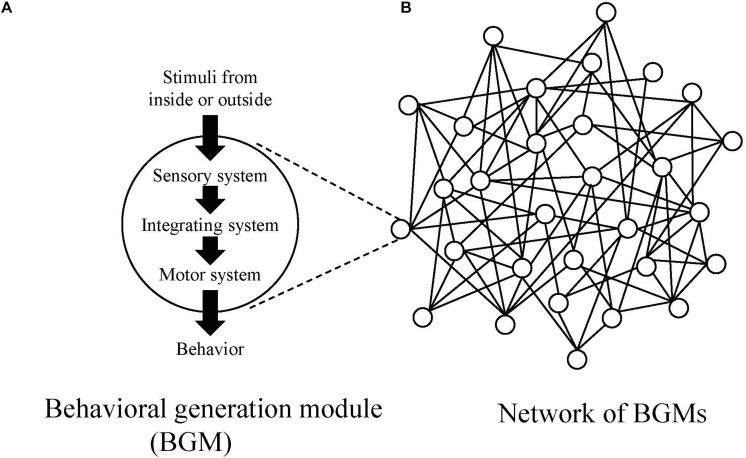
**(A)** Conceptual diagrams of behavioral generation module (BGM) and **(B)** the network of BGMs. In the network, circles represent BGMs, and lines represent links between BGMs.

In this paper, in order to investigate mechanism to generate behaviors for all animal species, the authors introduce a conceptual model, “behavioral generation module, BGM (Figure 1A).” BGM consists of the three systems (sensory, motor and integrating ones) and is responsible for generating behaviors ranging from simple to complex ones, such as reflex, taxis, kinesis, and modal action pattern (MAP) (Barlow, 1968). MAP is a stereotypic, species-specific behavioral pattern that is triggered by a specific stimulus. For example, the mating dance of male stickleback fish (Tinbergen, 1951) and web building of a spider (Risch, 1977).

In the case of animals with nervous system, BGM includes reflex arc, command neuron, central pattern generator (CPG) and “center and innate release mechanism (IRM)” proposed by Tinbergen (1951). In case of animals without neurons, for example, protozoan ciliate, physical mechanisms regulating electric phenomena in the membrane corresponds to BGM for its swimming behavior. The swimming of a ciliate is driven by the collective motion of many cilia, which is controlled by the membrane potential and the Ca^2+^ current (Kunita et al., 2016).

As an animal shows various behavioral repertoires, it is natural to assume the existence of BGMs. Additionally, because each actual BGM, such as CPG, forms connections with other ones through neural, hormonal, and physical pathways, one can also assume a complex network constituted by BGMs and links connecting them (Figure 1B).

### Potential and Activated BGMs

When an animal receives an appropriate stimulus, a BGM is activated and the animal executes a behavior. In the process, inside BGM, at first, the sensory system accepts and processes the stimulus. This is followed by the integration and interpretation of the input information by the integrating system, which then decides what kind of reaction to do. Finally, the motor system gives instructions in appropriate time series to motor organs such as muscles.

To activate a BGM, the BGM must be “ready to work” before accepting the stimulus. For example, if the BGM is a specific nervous system to generate a MAP, it must be “motivated” by appropriate drives and if the BGM is neurons of a CPG or membrane of a single cell organism, they must be “charged” at the appropriate level.

In this paper, the authors define the BGM that is activated by the appropriate stimulus, generating a behavior, as an “activated BGM.” Additionally, the authors define the BGM that is ready for generating a behavior before accepting the stimulus as a “potential BGM.” To understand the activated and potential BGMs more clearly, the authors explain them below by introducing turn alternation behavior in pill bugs.

#### Activated and Potential BGM: Turn Alternation in Pill Bugs

When animals move forward, the tendency to turn in the opposite direction of a preceding turn has been observed in a wide range of species. In terrestrial isopods such as pill bugs and sow bugs, this behavior has been studied extensively and assumed to be generated by internal responses to leg movements (Beal and Webster, 1971). The main hypothesis regarding the mechanism underlying this behavior is based primarily on proprioceptive information from the previous turn (Hughes, 1985).

The alternating turn behavior which is thought to be generated by response to such internal states is called turn alternation (Hughes, 1989). In pill bugs, turn alternation is considered as a MAP released by the preceding turn as a stimulus. The BGM for turn alternation is the internal mechanism that changes bilaterally asymmetrical leg movements that occur in the preceding turning (Hughes, 1985).

Besides the BGM for turn alternation, many other BGMs, e.g., for conglobation, eating, mating, and so on, are assumed. In the field, when pill bugs do not generate a specific MAP, they wander around on the ground, which is called “appetitive behavior” (Tinbergen, 1951), until they encounter an appropriate stimulus.

**FIGURE 2:**
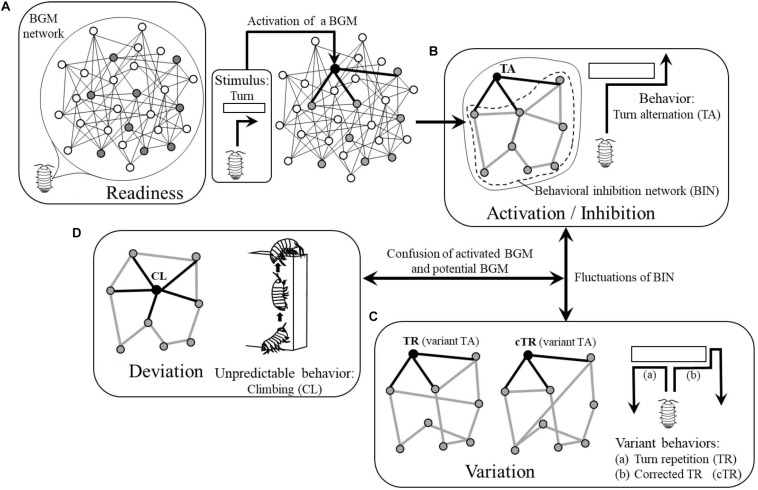
Four modes of BGM network: **(A)** Readiness. **(B)** Activation/Inhibition. **(C)** Variation. **(D)** Deviation, corresponding behaviors of pill bug as actual examples. White circles, resting BGMs; gray circles, potential BGMs; black circles, activated BGMs. Thin black lines, links between BGMs; thick black lines, links between an activated BGM and potential BGMs; thick gray lines, links forming behavioral inhibition network (BIN). See details in text.

During wandering, they will receive various stimuli as drives and some BGMs will be motivated. For example, if they receive vibration from the ground incidentally, the BGM for conglobation will be motivated and ready for generating the related behavior while they keep wandering (i.e., they will wander “nervously”). In this way, various BGMs are in the state of readiness to generate behaviors and are latent in the BGM network as “potential BGMs”’ until they receive appropriate stimuli (Figure 2A).

The wandering pill bug will, at some point, encounter an obstacle and be forced to turn right or left. If the BGM for turn alternation has been a potential BGM, it will be activated and generate a turn in the opposite direction of a preceding turn, i.e., execute turn alternation. In this way, the BGM for turn alternation becomes an “activated BGM” (Figure 2B).

### Behavioral Inhibition Network (BIN)

In this section, the authors will introduce the concept of a behavioral inhibition network. In nature, while executing a behavior, an animal receives various stimuli and potential BGMs are likely to be activated. For example, if the pill bug is turning at an obstacle of which the surface is covered with setae, such as the head of a toothbrush, it will also receive vibration from the setae and the potential BGM for conglobation will become ready for activation. To execute turn alternation, the activated BGM for turn alternation should send a command to the potential BGM for conglobation to inhibit generation of this behavior, otherwise turn alternation will be interrupted by the generation of conglobation behavior.

This example suggests that to generate and maintain a behavior, an activated BGM should send commands to all potential BGMs instructing them to inhibit generation of behaviors. However, because not all potential BGMs have direct links with the activated BGM (Figure 2B), only some of the potential BGMs receive these commands and therefore send them to the neighboring ones.

As stated above, the authors assume that all potential BGMs can inhibit generation of behaviors by sending commands to neighboring ones and as a result, will constitute a network where they inhibit generation of behaviors through each other. In this paper, the authors call the network where potential BGMs mutually inhibit their activities, “behavioral inhibition network (BIN) (Figure 2B).”

By inhibiting activities mutually, when an activated BGM generates a behavior, potential BGMs that do not have direct links with the activated BGM can suppress their activities. As a result, because the potential BGMs inhibit generation of behaviors, the animal can execute the necessary or desired behavior without being interrupted by erroneous behaviors. In the example of the pill bug’s behavior, it can execute turn alternation without being interrupted by conglobation, even when it encounters an obstacle of which the surface is covered with setae.

### Variation and Deviation of Behaviors

The authors assume that in a BIN, each potential BGM chooses one or more BGMs among neighboring ones at random in order to inhibit their activities; the structure of the BIN therefore changes from moment to moment (Figure 2C). In this way, the structure of a BIN is not static but dynamically maintained. The fluctuation of the structure of the BIN will be transmitted to the activated BGM through links and might affect its function. As a result, the behavior of the animal will be changed and will occasionally become variant behavior. For example, instead of tun alternation, the pill bug might turn in the same direction of a preceding turn (Figure 2Ca) or change the direction of the turn (Figure 2Cb).

In the process of dynamical maintenance of a BIN, in some rare cases, one of the potential BGMs might be confused with the activated BGM and generate the potential behavior (Figure 2D). In the case of the pill bug, for example, it might climb the obstacle where it should normally turn. As such climbing behavior often appears when pill bugs are in a humid situation in order to evaporate excess water from inside of their body (e.g., one can often observe that they climb the exterior walls of houses or fences after rain), this behavior is not adaptive to a normal dry situation. In this paper, the authors call such a behavior that deviates from the context of adaptation “unpredictable behavior (Figure 2D).”

### BIN as Animal Mind

As described above, on the one hand BIN is necessary to generate and maintain a behavior, while on the other hand it can deviate behavior from adaptive to unpredictable. This behavioral unpredictability results in observers feeling “unpredictability” of the animal. In this way, BIN forms “the source of unpredictability,” i.e., the essence of mind, inside an animal. What, then, is mind? Here, the authors answer that “It is BIN.”

Although the generation of unpredictable behaviors is the characteristic capacity of the mind, it is difficult to judge whether an unpredictable behavior results from incidental environmental change or the capacity of the mind. For example, the pill bug showing turn alternation may climb the obstacle suddenly because of incidental rapid rise of humidity. On the other hand, in an experiment where pill bugs were exposed to an unexperienced problematic situation, they climbed the wall of the apparatus in the normal dry condition and escaped from the situation (described in more detail below) (Moriyama, 1999). From this experimental result, the authors hypothesized that the capacity of the mind to generate unpredictable behaviors will be used to create emergent behaviors to survive in unexperienced situations.

In the section “Innate and Emergent Behavior of Animals,” the authors introduce some experimental results to support the BIN hypothesis. These results report that there is a relationship between fluctuation of adaptive behaviors and creation of emergent behaviors in unexperienced situations.

## Innate and Emergent Behavior of Animals

It has been considered that species-specific behavioral patterns such as MAP (Barlow, 1968) have been acquired through various processes of evolution, mainly natural selection (e.g., Eibl-Eibesfeldt, 1970). One can expect that such species-specific behaviors will be elicited in response to the same stimuli (e.g., Eibl-Eibesfeldt, 1970). Species-specific behaviors should be stable if the mental capacity of focal animals were low; animals with poorer mental capacities will choose constant behavioral patterns in a wider range of environmental conditions. Animals with higher mental capacities, on the other hand, may choose and/or regulate their behavior patterns according to changes in their environments. Several authors have suggested that humans can choose behaviors other than species-specific ones (e.g., Lorenz, 1973; Dawkins, 1976).

Animals with considerably high mental capacity may show emergent behaviors in such situations where innate behaviors are likely to be released but destined to be disadvantageous; the observer may view this as the existence of mind. Thus, if this kind of ability to produce behavioral patterns overcoming innate constraint is observed, one may think the animal has mind, even though its nervous system is relatively simple, and its mental capacity seems to be poor. Here, one can see that the observers’ concept for animal mind shifts from mental capacity to the capacity of creating emergent behavior, i.e., the function of BIN.

The following examples may demonstrate that mind is more ubiquitous than expected by many researchers of comparative psychology. In the experiments introduced below, unexperienced problematic situations, where an ancestor of the species might not have been involved, were provided experimentally; the animals showed emergent behaviors to survive in these situations. In each experiment, the author(s) proposed original and valid mechanisms generating the emergent behavior. The authors of this paper suggest that BIN underlies these mechanisms.

### Hermit Crabs

Gunji (1996) reported behavioral plasticity in a hermit crab *Coenobita purpureus*. In his preliminary observations, Gunji found that hermit crabs seldom came out from their shells voluntarily and tended to proceed when pulled backward by their shells. Hermit crabs without their own shells were vulnerable to cannibalism. Gunji devised an experimental setting where only pairs of two shells attached back to back were available. He observed two types of emergent group behaviors. One was that pairs of individuals abandoned the shells and walked around without going into cannibalism. The other is that two individuals shared shells attached back to back and developed a cooperative walk; that is, when one of paired individuals stepped forward, the other stepped backward, and vice versa.

### Gobiid Fish and Snapping Shrimps

**FIGURE 3:**
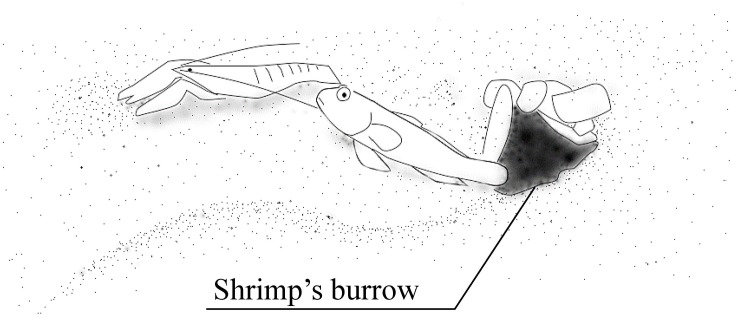
Goby-shrimp symbiosis. The goby utilizes the burrow dug by the snapping shrimp as its nest and shelter. The fish informs the shrimp of the predators’ approach by quivering its body. The shrimp is always touching the fish with its antenna when it comes out of the burrow.

Migita and Gunji (1996) showed plasticity in the symbiotic behavior of the gobiid fish *Amblyeleotris steinitzi*. Symbiosis between gobiid fish and snapping shrimps is a well-known example of a mutually beneficial relationship, where the fish use the shrimps’ burrows as shelters and in turn, informs the shrimps of approaching predators (Figure 3). Gobiid fish can visually perceive predators much better than snapping shrimps, who have rather poor visual capacity. When a potential predator is approaching the gobiid fish and its partner shrimp is touching the fish with its antennae, it quivers its tail until the shrimp stops moving and/or retreats into the shrimp’s burrow. The gobiid fish retreats into the burrow if the predator approaches further. Thus, the gobiid fish uses burrows of symbiotic snapping shrimps as a shelter when predatory fish approach, as well as nests. It does not enter burrows of other animals unless it is in emergency.

**FIGURE 4:**
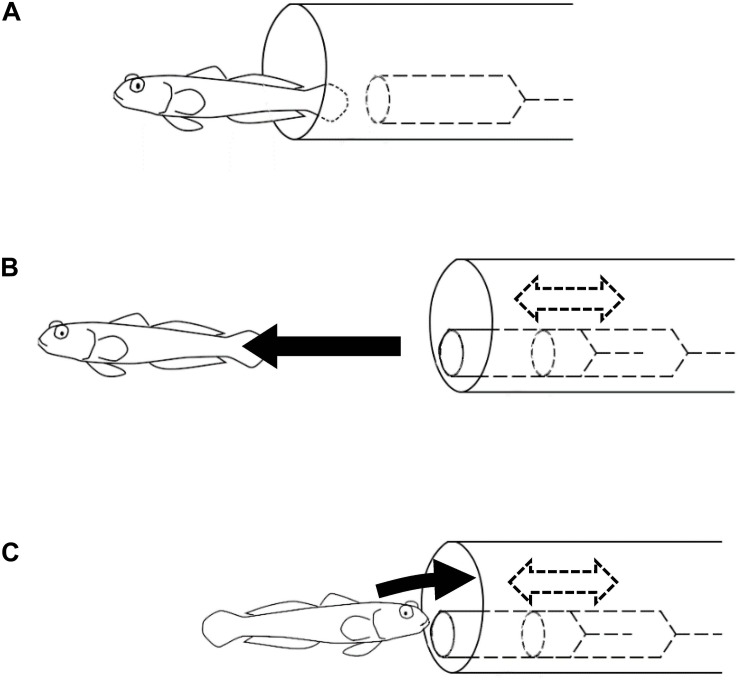
Experiments on the symbiotic behavior. Black arrows represent movements of the goby. Dashed arrows and lines represent movements and objects inside the artificial burrow. **(A)** Soon after the goby immigrated in the experimental tank, it started to use the artificial burrow containing a motionless model shrimp. **(B)** In the earlier trials, the goby swam away from the artificial burrow as the model shrimp was moved. **(C)** Some of the subject fish got used to retreating into the artificial burrow in response to the same operation of the model shrimp.

The symbioses between gobiid fish and snapping shrimps are mutually beneficial and considered to have evolved through natural selection. In the natural habitat, *A. steinitzi* has been observed as associated with snapping shrimps. However, in the experimental environment without a snapping shrimp but rather a model of a shrimp in an artificial burrow, *A. steinitzi* could learn to use the artificial burrow with the model shrimp as a shelter. Such individuals acquired “emergent symbiotic behavior” somewhat different from that observed in their natural habitat. In the natural symbiotic behavior of the gobiid fish, they warn the snapping shrimps of predators’ existence, i.e., the snapping shrimp stays at the end of the burrow. On the other hand, in the “emergent symbiotic behavior,” the gobiid fish retreated into the artificial burrow when the model shrimp came out from the burrow (Figure 4).

### Ants

Kitabayashi et al. (1999) investigated food transportation by the ant *Formica japonica* and discussed that the emergence of novel behavior should involve statistical properties called 1/f noise and/or Zipf’s law (Zipf, 1949). In their experiments on the ant’s foraging, they prepared various kinds of food with different levels of preference. When grains of a preferred food were put on a sheet of less preferred food, that was too large for an ant to transport, the ants tended to collect the grains of preferred food. However, the ants sometimes exhibited emergent cooperative transportation of the sheet of less preferred food with more preferred food on top. They called this emergent behavior, tool transportation. Their analyses on the time series of number of foragers indicated a strong relationship between emergent change of mode of behavior and such statistical properties of 1/f noise and/or Zipf’s law.

### Pill Bugs

**FIGURE 5:**
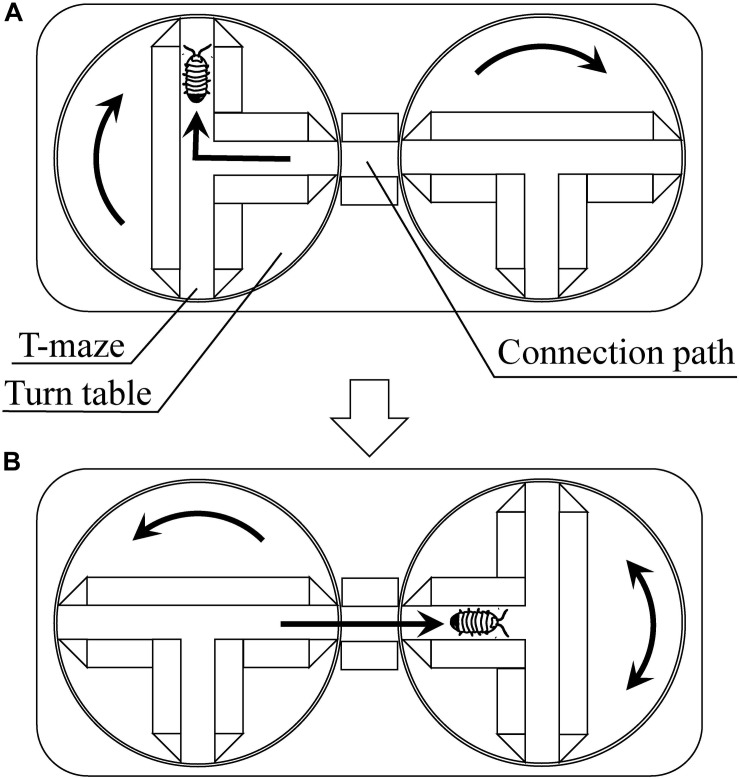
Multiple T-maze devise using turn tables [reprinted from Moriyama (2011)]. **(A)** When the pill bug turns to the right, the experimenter turns the maze to the right. Shortly after that, the experimenter turns another maze to the right to connect its central passage to the connection path. **(B)** Then, the pill bug passes through the connection path and reaches the central passage of the next maze. The bug will turn to the right or left, and the experimenter turns the maze to the same direction that the bug turned. Shortly after that, the experimenter turns another maze to the left. By repeating these procedures, the apparatus continuously gives the subject T-maze task.

Moriyama (1999, 2004) reported on plasticity inherent in the fixed behavioral pattern known as “turn alternation” and the emergent problem-solving behavior. As discussed earlier in the paper, turn alternation is a behavioral pattern in which a pill bug turns left and right alternately when it encounters an obstacle. This behavior has been considered to be advantageous as it enables an escaping pill bug to take a relatively straight route in environments with many obstacles. Moriyama (1999) investigated the pill bug’s behavior with T-mazes on turntables (Figure 5). In his experiments, pill bugs were tested 200 times through successive T-choices. In this experimental setting, turn alternation cannot contribute to the escape from the T-mazes. Under this unexperienced problematic situation, some pill bugs repeated increase and decrease of the success rate of turn alternation, i.e., fluctuated turn alternation, and finally, climbed up the walls surrounding the maze and escaped from the experimental apparatus. This climbing behavior can be considered as emergent escaping behavior.

**FIGURE 6:**
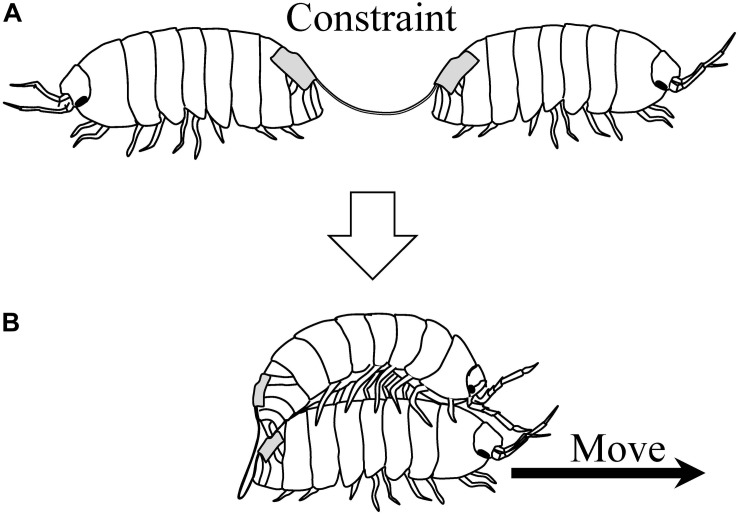
**(A)** Pill bugs connected back-to-back with a string. **(B)** Emergent cooperative movement of the pair [reprinted from Moriyama (2011)].

Moriyama (2004) later investigated the behavior of pairs of pill bugs connected back-to-back with a string (Figure 6A). Thus, pill bugs in a pair were constrained in their movements. Normally, they would persist in walking forward when pulled backward. However, some pairs showed cooperative movements, in a sense, when they were released into an experimental arena for 3 h. Some pairs moved by one individual mounting on the other’s back (Figure 6B); other pairs demonstrated moving by a death-feigning individual being pulled by the other. Thus, pairs exhibiting the novel behaviors could move around the arena, despite the string constraining the individual pill bug’s movements. These behaviors are considered as emergent cooperative movement. In this experiment, as a visible index of fluctuation of their behavior, the time sequential changing of distance between individual pill bugs were analyzed. As a result, Zipf’s law was observed in the frequency distribution of the distance of the pairs showing emergent cooperative movement.

## Discussion

In some of the studies presented, the authors found common and interesting results. In the individuals that generated emergent behavior, for example, a power law was observed in the fluctuation of behavior until the emergent behavior appeared (see the examples of ants and pill bugs in section “Innate and Emergent Behavior of Animals”). Power law is known to be observed in the process of self-organization of various spatiotemporal structures in nature (Bak, 1996). The power law that appeared in the fluctuation of behavior implies that this fluctuation is endogenous. The correlation between the endogenous fluctuation and the generation of emergent behavior suggests that the generation mechanisms of both phenomena are related or the same. This result supports the authors hypothesis that BIN is responsible for generating both behavioral fluctuations and unpredictable behaviors.

Mind has been defined by different theoretical models in psychology. For instance, behaviorism considers that the mind is composed by stimulus-response associations created with learning (e.g., Watson, 1913; Skinner, 1950), cognitivism does that the mind is composed by processes that transform input information, and create schemas (e.g., Neisser, 1967). In more recent approaches, for example, the one based on the free-energy principle considers that the mind arises from the brain’s tendency to best predict the environment, i.e., the incoming information, to save energy consumption (Friston, 2010). In all these formulations, unlike our BIN hypothesis, producing emergent behaviors does not count as having a mind.

What the authors want to suggest is that observing unpredictable, emergent behaviors in other animals may give us the impression of observing mind, while this may be simply the result of the activity of the dynamical maintenance of the BIN.

The BIN hypothesis described in section “Animal Mind as a Behavioral Inhibition Network” of the paper is as follows: (1) Behavioral fluctuations are generated by the activity of the dynamical maintenance of BIN affecting the activities of the activated BGM; (2) Unpredictable behavior is generated by confusion of the potential BGM and the activated BGM accompanying the activity of the dynamical maintenance of the BIN. The authors considered that animals can self-adjust the probability of confusion and increase the incidence of unpredictable behavior when tackling unexperienced problematic situations.

The authors thus proposed a meaning of mind in daily life as the source of unpredictability and concluded that it can exist as BIN. Suppose one is asked, “Do you have mind?” Many people will answer immediately, “Of course.” One is then asked, “What is mind?” At this question many people will be hesitant to answer. In this way, mind is “something indefinite” that allows one to be convinced of the existence inside of themselves, yet unable to explain its substance.

Behavioral inhibition network matches this “something indefinite” explanation. As mentioned in section “Animal Mind as a Behavioral Inhibition Network” of this paper, the structure of BIN is maintained dynamically and does not keep a certain shape. Additionally, its function is also undefined because, as already mentioned, while supporting the generation and maintenance of adaptive behaviors, it suddenly contributes to generate unpredictable behaviors.

Thus, to answer the question, “What is mind?”; the authors propose that it is BIN. Thus, the source of unpredictability that one feels when facing animals, as well as its presence inside us is mind and the substance of mind is BIN.

The authors believe that mind as BIN-like structure exists not only in animals but also in plants and materials such as stones. At present, the authors are exploring experimental methods to derive emergent activities from plants and other materials. The authors present a few examples of research that suggest the possibility of the generation of emergent behaviors in plants below.

Plants such as grass and trees that one usually imagines are generally composed of a stem that is the main axis, branches that branch from it, leaves that grow on the stem and branches, and underground roots. If one was to look closely at the stems and branches of the main axis, one could see that both are composed of a unit consisting of a single stem and the leaves that grow on it. This unit is called “shoot” in botany. The ground part of grass and trees that one usually sees is in fact a “shoot system” composed of many shoots.

Each shoot has the capacity to grow independently. There are various repertories in this growth such as extension of the tip, formation of infant shoots (lateral buds) and generation of young leaves and roots. Therefore, in a plant individual, each shoot is considered to be a BGM that generates various growth patterns as behaviors. In fact, there is a proposal in which a unit that is similar in shape to a shoot and that constitutes a plant body by repetition is called a module (Harper, 1985). Moreover, this modularity is considered to be one of the factors of high plasticity in plants (de Kroon et al., 2005).

If a plant is considered to be a collection of BGMs, when a certain plant behaves as one individual, such as growing toward a strong light source, several shoots grow as activated BGMs and some other ones suppress their growth as potential BGMs. The latent BGMs will then form BIN. In fact, each part of a plant individual forms a network that transmits its state to other parts using electricity, chemicals, and water (Mancuso and Viola, 2016).

One method confirming autonomous suppression of the shoot growth in BIN is trimming, which is well known in horticulture. When a shoot of a plant is cut out and planted in the soil, the shoot fragment generates roots, and becomes a new plant individual if the conditions are appropriate. One usually does not see roots growing from the stems of plants. This is because each shoot autonomously suppresses potential root growth.

The question that arises is, can the mind of plants, that is, the shoots that make up BIN, exhibit emergent behavior in an unexperienced situation? One example of this ability is the behavior of a host plant parasitized by the parasitic plant called dodder (*Cuscuta* spp.) (Hettenhausen et al., 2017).

The dodder is a vine parasitic plant. When it reaches the host plant by extending the vine, it wraps around the body, inserts the parasitic root into the host vascular bundle, and absorbs water and nutrients. In reported experiments, several plants were bridged by dodders. When one of these host plants is fed by an insect, and a systemic signal is transmitted from the damaged plant to the non-affected plant, which can prepare a defensive response against the insect. The dodder has the negative effect of looting nutrients from the host plant, while the host plant is able to use the long vine of the dodder as a novel stem.

In host plants, the dodder parasitism is an unexperienced situation of continuous feeding damage, unlike temporary damage caused by animal feeding, for example. Shoots with inserted parasitic roots usually suppress the ability to grow new stems and roots freely. On the other hand, in the unexperienced state of parasitism, it is considered that the plant generated emergent growth through assimilating the new stem into the external structure of the dodder vine. In fact, when examining the mRNA of parasitic roots inserted into host plants, it was reported that half of them were derived from the host (Kim et al., 2014). This report suggests that the host plant tried to assimilate its stem with the vine of the dodder.

If one can assume mind for plants with little motion, one may be able to assume mind even for materials that do not move like stones. The following study suggests that stones have at least BGM-like module.

European stone craftsmen smash rock stones called flint repeatedly with hammer stones to make stone tools. With experience, they can break a flake of the desired shape by applying the minimum necessary force to a specific location on the stone (Nonaka et al., 2010). That is, flint stones produce specific shaped flakes when a certain amount of force is applied to a specific location. This phenomenon is the same as one in which a pill bug turns left and then turns right.

A flint stone is thus considered to include BGM-like modules, each of which generates a specific shape of a flake by stimulating a specific magnitude of striking force at an arbitrary hit point. These BGM-like modules that make up an individual stone will form a network, much like the example of the shoot. Thus, if a stone also has BIN-like structure, as is suggested by this study, it will generate emergent phenomenon in an unexperienced situation. The authors are currently investigating how to give an unexperienced situation to stones and objects such as glass to derive emergent phenomena from them.

## Limitations and Concluding Remarks

In this paper, the authors proposed an additional new perspective that the impression of mind that one usually has in our daily life is unpredictability and its source is BIN. BIN is a promising candidate for the source of endogenous fluctuations widely observed in animal behavior. In addition, this ability to generate behavioral fluctuation, as in the experimental example introduced in section “Innate and Emergent Behavior of Animals,” generates emergent behavior in an unexperienced situation and helps animals to survive.

Although the authors illustrated emergent behaviors in a wide range of animals, it was limited to “simple” animals. Further studies on complex animals such as mammals, including human beings, are needed to verify the universality of BIN.

Approaching the substance of BIN not only reveals that mind as the source of unpredictability exists in all living things and materials, but also brings a new worldview that all living things and materials have creativity of the generation of emergent phenomena.

## Author Contributions

TM, KS, HS, and MM contributed to the conception and design of the study, and wrote sections of the manuscript. TM and KS wrote the first draft of the manuscript. All authors contributed to the manuscript revision, read, and approved the submitted version.

## Conflict of Interest

The authors declare that the research was conducted in the absence of any commercial or financial relationships that could be construed as a potential conflict of interest.

## References

[B1] Bak P. (1996). *How Nature Works? The Science of Self-Organized Criticality.* New York, NY: Springer.

[B2] Barlow G. W. (1968). “Ethological units of behaviour,” in *The Central Nervous System and Fish Behavior*, ed. Ingle D. J. (Chicago: University of Chicago Press), 217–232.

[B3] Beal I. L. Webster D. M. (1971). The relevance of leg-movement cues to turn alternation in woodlice (*Porcellio scaber*). *Anim. Behav.* 19 353–356. 10.1016/s0003-3472(71)80016-7

[B4] Cambridge dictionary (2019). Available online at: https://dictionary.cambridge.org/dictionary/english/mind/ (accessed October 1, 2019).

[B5] Darwin C. R. (1871). *The Descent of Man and Selection in Relation to Sex.* New York, NY: D. Appleton and Company.

[B6] Dawkins R. (1976). *The Selfish Gene.* Oxford: Oxford University Press.

[B7] de Kroon H. Huber H. Stuefer J. F. van Groenendael J. M. (2005). A modular concept of phenotypic plasticity in plants. *New Phytol.* 166 73–82. 10.1111/j.1469-8137.2004.01310.x 15760352

[B8] Delcomyn F. (1998). *Foundations of Neurobiology.* New York, NY: Freeman.

[B9] Eibl-Eibesfeldt I. (1970). *Ethology - The Biology of Behavior.* New York, NY: Holt, Rinehart and Winston.

[B10] Fiorito G. Scotto P. (1992). Observational learning in *Octopus vulgaris*. *Science* 256 545–547. 10.1126/science.256.5056.545 17787951

[B11] Friston K. (2010). The free-energy principle: a unified brain theory? *Nat. Rev. Neurosci.* 11 127–138. 10.1038/nrn2787 20068583

[B12] Gunji Y. P. (1996). Behavioral plasticity of hermit crabs-A preliminary report. *Riv. Biol.- Biol. Forum* 89 69–78.

[B13] Harper J. L. (1985). “Modules, branches, and the capture of resources,” in *Population Biology and Evolution of Clonal Organisms*, eds Jackson J. B. C. Buss L. W. Cook R. E. (New Haven: Yale University Press), 1–34. 10.2307/j.ctt2250w9n.4

[B14] Hettenhausen C. Li J. Zhuang H. Sun H. Xu Y. Qi J. (2017). Stem parasitic plant *Cuscuta australis* (dodder) transfers herbivory-induced signals among plants. *PNAS* 114 E6703–E6709. 10.1073/pnas.1704536114 28739895 PMC5559024

[B15] Hinde R. A. (1982). *Ethology: Its Nature and Relations with Other Sciences.* New York, NY: Oxford University Press.

[B16] Hughes R. N. (1985). Mechanisms for turn alternation in woodlice. *Anim. Learn. Behav.* 13 253–260. 10.1016/0376-6357(87)90069-6 24896868

[B17] Hughes R. N. (1989). “Phylogenic comparison,” in *Spontaneous Alternation Behavior*, eds Dember W. N. Richman C. L. (New York, NY: Springer), 39–57.

[B18] Hunt G. R. (1996). Manufacture and use of hook-tools by new caledonian crows. *Nature* 379 249–251. 10.1038/379249a0

[B19] Kim G. LeBlanc M. L. Wafula E. K. dePamphilis C. W. Westwood J. H. (2014). Genomic-scale exchange of mRNA between a parasitic plant and its hosts. *Science* 345 808–811. 10.1126/science.1253122 25124438

[B20] Kitabayashi N. Kusunoki Y. Gunji Y. P. (1999). The emergence of the concept of a tool in food-retrieving behavior of the ants *Formica japonica* (Motschulsky). *BioSystems* 50 143–156. 10367976 10.1016/s0303-2647(98)00096-3

[B21] Kojien (2018). *Kojien*, 7th Edn Tokyo: Iwanami Shoten.

[B22] Kunita I. Yamaguchi T. Tero A. Akiyama M. Kuroda S. Nakagaki T. (2016). A ciliate memorizes the geometry of a swimming arena. *J. Royal Soc. Interface* 13:20160155. 10.1098/rsif.2016.0155 27226383 PMC4892268

[B23] Lorenz K. (1973). *Die Rückseite des Spiegels.* München: Piper Verlag.

[B24] Mancuso S. Viola A. (2016). *Brilliant Green: The Surprising History and Science of Plant Intelligence.* Washington DC: Island Press.

[B25] Migita M. Gunji Y. P. (1996). Plasticity in symbiotic behavior: as demonstrated by a gobiid fish (*Amblyeleotris steinitzi*) associated with alpheid shrimps. *Riv. Biol. Biol. Forum* 89 389–406.

[B26] Migita M. Mizukami E. Gunji Y. P. (2005). Flexibility in starfish behavior by multi-layered mechanism of self-organization. *BioSystems* 82 107–115. 10.1016/j.biosystems.2005.05.012 16214287

[B27] Moriyama T. (1999). Decision-making and turn alternation in pill bugs. *Int. J. Comp. Psychol.* 12 153–170. 10.1016/j.beproc.2015.11.016 26621257

[B28] Moriyama T. (2004). Problem solving and autonomous behavior in pill bugs (*Armadillidium vulgare*). *Ecol. Psychol.* 16 287–302. 10.1207/s15326969eco1604_2

[B29] Moriyama T. (2011). *Is There Mind in Pill Bugs?.* Tokyo: PHP Institute.

[B30] Nakagaki T. Yamada H. Tóth Á (2000). Maze-solving by an amoeboid organism. *Nature* 407:470. 10.1038/35035159 11028990

[B31] Nakagaki T. Yamada H. Tóth Á (2001). Path finding by tube morphogenesis in an amoeboid organism. *Biophys. Chem.* 92 47–52. 10.1016/s0301-4622(01)00179-x 11527578

[B32] Neisser U. (1967). *Cognitive Psychology.* New York, NY: Appleton-Century-Crofts.

[B33] Nonaka T. Bril B. Rein R. (2010). How do stone knappers predict and control the outcome of flaking? Implications for understanding early stone tool technology. *J. Hum. Evol.* 59 155–167. 10.1016/j.jhevol.2010.04.006 20594585

[B34] Papini M. R. (2002). *Comparative Psychology: Evolution and Development of Animal Behavior 1st Edition.* Upper Saddle River, NJ: Prentice Hall.

[B35] Pepperberg I. M. (2002). *The Alex Studies: Cognitive and Communicative Abilities of Grey Parrots.* Cambridge, MA: Harvard University Press.

[B36] Reese E. S. (1996). “The complex behavior of echinoderms,” in *Physiology of Echinodermata*, ed. Boolootian R. A. (New York, NY: Interscience Publishers), 157–218.

[B37] Risch P. (1977). Quantitative analysis of orb-web patterns in four species of spiders. *Behav. Genet.* 7 199–238. 10.1007/bf01066276 869859

[B38] Skinner B. F. (1950). Are theories of learning necessary? *Psychol. Rev.* 57 193–216. 10.1037/h0054367 15440996

[B39] Tinbergen N. (1951). *The Study of Instinct.* New York, NY: Oxford University Press.

[B40] Watson J. B. (1913). Psychology as the behaviorist views it. *Psychol. Rev.* 20 158–177. 10.1037/h0074428

[B41] Yoshida M. Hirano R. (2009). Photocardiography: a novel method for monitoring cardiac activity in fish. *Zool. Sci.* 26 356–361. 10.2108/zsj.26.356 19715506

[B42] Zipf G. K. (1949). *Human Behavior and The Principal of Least Effort.* Cambridge, MA: Addison-Wesley.

